# Sex Differences in the Cognitive and Hippocampal Effects of Streptozotocin in an Animal Model of Sporadic AD

**DOI:** 10.3389/fnagi.2017.00347

**Published:** 2017-10-31

**Authors:** Jian Bao, Yacoubou A. R. Mahaman, Rong Liu, Jian-Zhi Wang, Zhiguo Zhang, Bin Zhang, Xiaochuan Wang

**Affiliations:** ^1^Key Laboratory of Ministry of Education of China for Neurological Disorders, Department of Pathophysiology, School of Basic Medicine and the Collaborative Innovation Center for Brain Science, Tongji Medical College, Huazhong University of Science and Technology, Wuhan, China; ^2^Co-innovation Center of Neuroregeneration, Nantong University, Nantong, China; ^3^School of Medicine and Health Management, Tongji Medical College, Huazhong University of Science and Technology, Wuhan, China; ^4^Department of Genetics and Genomic Sciences, Icahn School of Medicine at Mount Sinai, New York, NY, United States

**Keywords:** Alzheimer's disease (AD), animal model, Streptozotocin (STZ), sex differences, learning and memory

## Abstract

More than 95% of Alzheimer's disease (AD) belongs to sporadic AD (sAD), and related animal models are the important research tools for investigating the pathogenesis and developing new drugs for sAD. An intracerebroventricular infusion of streptozotocin (ICV-STZ) is commonly employed to generate sporadic AD animal model. Moreover, the potential impact of sex on brain function is now emphasized in the field of AD. However, whether sex differences exist in AD animal models remains unknown. Here we reported that ICV-STZ remarkably resulted in learning and memory impairment in the Sprague-Dawley male rats, but not in the female rats. We also found tau hyperphosphorylation, an increase of Aβ40/42 as well as increase in both GSK-3β and BACE1 activities, while a loss of dendritic and synaptic plasticity was observed in the male STZ rats. However, STZ did not induce above alterations in the female rats. Furthermore, estradiol levels of serum and hippocampus of female rats were much higher than that of male rats. In conclusion, sex differences exist in this sporadic AD animal model (Sprague-Dawley rats induced by STZ), and this should be considered in future AD research.

## Introduction

Alzheimer's disease (AD) is one of the most common neurodegenerative diseases, affecting about 35 million people all over the world. And its prevalence is expected to reach 115 million by 2050 due to aggravating trend of aging population, unless there are available treatments that can prevent or cure this disease (Mangialasche et al., 2010). Therefore, an appropriate animal model is an important research tool for finding valid AD treatments. Yet, over the last 20 years, many of the potential drugs that target tau and Aβ, the two hallmarks of AD, failed in clinical trials, though some of treatments were effective in AD animal models (Zahs and Ashe, 2010; Shineman et al., 2011; Hall and Roberson, 2012). Thus, it is crucial to re-evaluate the existing animal models of AD.

AD exists mainly in two forms: familial (fAD) and sporadic (sAD). More than 95% of cases belong to sAD, for which aging and metabolic disorders are the main non-genetic risk factors (Kloppenborg et al., 2008). Intracerebroventricular streptozotocin (ICV-STZ) injection produces cognitive deficits in rats, as well as cholinergic dysfunction, tau hyperphosphorylation, insulin receptor dysfunction, impaired energy metabolism, and oxidative stress (Hong and Lee, 1997; Prickaerts et al., 1999; Salkovic-Petrisic and Hoyer, 2007; Deng et al., 2009). These changes are similar to those observed in the brain of patients with sporadic AD. Therefore, ICV-STZ treated rats have been proposed as a research model of sAD (Lannert and Hoyer, 1998; Mehla et al., 2013). Meanwhile, ICV-STZ animal model has been used to evaluate the therapeutic potential of numerous old and novel drugs and compounds, as well as other non-drug therapies (Jee et al., 2008; Rodrigues et al., 2010; Salkovic-Petrisic et al., 2013). Nevertheless, although effectiveness of the therapeutic strategies has been proved in ICV-STZ model, the therapies failed to achieve similar therapeutic effects on learning and memory deficits in sAD clinical trials, like those with NSAIDs and PPAR agonists or vitamin E and Ginkgo biloba (Woo, 2000; Salkovic-Petrisic et al., 2013; Dysken et al., 2014; Malkki, 2016; Prasad, 2017; Wightman, 2017). Thus, it is necessary to characterize and re-evaluate the ICV-STZ animal model.

Accumulating evidence indicates that there are some differences in structure, development, enzyme activity, and chemistry of the central nervous system (CNS) between female and male mammals (Becker et al., 2005; Cahill, 2006; McCarthy, 2009; Raznahan et al., 2010; Ruigrok et al., 2014; Forger et al., 2016). AD is one of major chronic neurodegenerative disorders that is histopathologically characterized by the intracellular neurofibrillary tangles (NFTs) that are composed of abnormally hyperphosphorylated tau and extracellular senile plaques that are accumulated of insoluble β-amyloid (Aβ), which result in a progressive cognitive impairment (Grundke-Iqbal et al., 1986; Alafuzoff et al., 1987). Although, it has been reported that ICV-STZ induces AD-like pathological changes, however, whether ICV STZ-induced sporadic AD in animal model is stable and universal in different sexes has not been reported. Here, we found that ICV-STZ remarkably induced AD-like pathological changes, including impaired learning and memory capacities; loss of dendritic and synaptic plasticity; tau hyperphosphorylation; increase in Aβ40/42 and increase in both GSK-3β and BACE1 activities in the male but not female STZ treated rats. Our study implies that sex difference should be taken into account during experiments design, results interpretation and drawing conclusions in AD research.

## Materials and methods

### Chemicals and antibodies

STZ was from Sigma (Sigma, St. Louis, MO, USA). Antibodies employed in this study are listed in Supplementary Table 1.

### Animal experiments

Two-month-old male (*n* = 24) and female (*n* = 24) Sprague-Dawley (SD) rats were provided by the Experiment Animal Center of Tongji Medical College, Huazhong University of Science and Technology. All animal experiments were performed according to the “Policies on the Use of Animals and Humans in Neuroscience Research” approved by Society for Neuroscience in 1995 and approved by the Experiment Animal Center of Tongji Medical College, Huazhong University of Science and Technology. The animals were individually housed in cages (house temperature 24°C, controlled humidity 40% and 12/12 h inverted light cycle) with free access to water and food.

STZ, soluble in artificial cerebrospinal fluid (aCSF), was injected slowly (1 μl/min) into the ventriculus lateralis cerebri of rats (10 μl, 3 mg/kg). Control animals were identically treated with the same volume of aCSF. After 30 days, morris water maze was employed to train and test spatial learning and memory. After this procedure which lasted for 7 days, mice were sacrificed and other tests were proceeded (Supplementary Figure 1).

### Morris water maze assay

The water maze used was a circular, steel pool (1.6 m in diameter) that was filled with black water (temperature 25°C) that was non-toxic and contrast to rat. A black-colored, circular platform (12 cm in diameter) was placed below the water surface at a specific location. Distinctive visual cues were stuck to the wall. For spatial training, rats were subjected to 4 trials each day from 2:00 to 5:00 p.m. The training was lasted 6 days and 24 trials were given to every rat. For each trial, the rat was placed at different starting position spaced equally around the perimeter of the pool. Rats were allowed to find the submerged platform within 60 s. If the rat could not find the hidden platform, it was then gently guided to the platform and allowed to stay there for 30 s. The time that each rat took to reach the platform was recorded as the escape latency. For the probe trial test, rats were submitted to the same pool with the platform removed and a probe trial of 60 s was given. The number of crossings and the time in the target quadrant were recorded.

### Western blotting

The protocol was performed as previously described (Xu et al., 2014). Four left hippocampus per group for Western blotting. Hippocampi were rapidly dissected out and homogenized in a buffer containing NaF 50 mM, Tris-Cl (pH 7.6) 10 mM, 1 mM EDTA, 1 mM Na_3_VO_4_, 1 mM benzamidine, and 1 mM phenylmethylsulfonylfluoride (PMSF), 10 g/ml leupeptin, and 2 g/ml each of pepstatin A and aprotinin. The homogenates were added to one-third of sample buffer containing 200 mM Tris-HCl (pH 7.6), 8% sodium dodecyl sulfate, and 40% glycerol, boiled in a water bath for 10 min, and then centrifuged at 14,510 r for 10 min. Protein concentration of the supernatants were measured by the bicinchoninic acid Protein Assay Kit (Pierce, Rockford, IL, USA). Ten micrograms of protein for DMIA and pS396 antibodies, 20 μg protein for other antibodies, were loaded and separated by SDS-polyacrylamide gel electrophoresis (10% gel), and then transferred to a nitrocellulose membrane. After blocking in 3% non-fat milk for 1 h, the nitrocellulose membranes were incubated with primary antibodies at 4°C overnight. The membranes were then incubated with secondary antibodies conjugated to IRDye (800CW) for 1–2 h and visualized using the Odyssey Infrared Imaging System (LI-Cor Biosciences, Lincoln, NE, USA). Image J software was employed for the quantitative analysis of the western blots.

### Golgi staining

The Golgi staining protocol was performed as previously described (Morest, 1960). Three per group were used for Golgi Staining. The rats were anesthetized with 6% chloral hydrate and perfused with 300 ml of normal saline containing 0.5% sodium nitrite, followed by 400 ml of 4% formaldehyde solution and further by ~400 ml dying solution (4% formaldehyde, 5% potassium dichromate, and 5% chloral hydrate) for 5 h in the dark. The brains were removed and incubated in the same fixative in the dark. After 3 days, the brains were transferred to a solution containing 1% silver nitrate for 3 days in the dark. The silver solution was changed each day. Thirty-five micrometers of thick coronal brain sections were cut using a vibrating microtome (Leica, VT1000S, Germany).

### Immunofluorescence

Three per group were used for Immunofluorescence Staining. The anesthetized rats were immediately perfused through the aorta with 300 ml normal saline, followed by a 300 ml solution containing 4% paraformaldehyde. The brains were dissected and post-fixed in 4% paraformaldehyde for another 48 h. Coronal sections (30 μm thick) were cut using a vibrating microtome. After incubation in 0.3% Triton-X100-PBS for 30 min at room temperature, free floating sections were blocked with 5% goat serum in PBS for 45 min at room temperature. Sections were then incubated overnight at 4°C with primary antibodies: polyclonal anti-MAP2 antibodies obtained from Abcam, (dilution 1:200, Cambridge, MA, USA). This was followed by incubation with secondary antibodies for 2 h at room temperature. The antibody staining was semi-quantitated by mean fluorescence intensities (MFIs) with Image J software.

### BACE1 enzymatic assay

The protocol was performed as previously described (Qi et al., 2016). Five right hippocampus per group for the assay. Beta-secretase activity was monitored using a commercial kit, from Abnova (Neihu District, Taipei City 114 Taiwan) according to the manufacturer instructions and using a multi-well fluorescence plate reader capable of Ex = 335–355 nm and Em = 495–510 nm. In briefly, 50 μl of 4 μg/μl hippocampus lysate was added to a 96-well plate. Fifty microliters of 2×reaction buffer were added, followed by 2 μl of β-secretase substrate. The reaction mixtures were incubating for 1 h in the dark. Fluorescence was monitored at excitation wave (wavelength = 334–355 nm) and emission wave (emission wavelength = 490–510 nm). β-secretase activity can be expressed as the Relative Fluorescence Units (RFU) per μg of protein sample.

### ELISA quantification of Aβ

The protocol was performed as previously described (Zhang et al., 2015). Five right hippocampus per group for the Elisa assay. To detect the concentration of Aβ in hippocampi lysates, the rat hippocampi were homogenized in buffer (PBS with 5% BSA and 0.03% Tween-20, supplemented with protease inhibitor cocktail), and centrifuged at 16,000 g for 20 min. Aβ1-40 or Aβ1-42 was quantified using the rats Aβ1-40 or Aβ1-42 ELISA Kit (Elabscience, Wuhan, China) in accordance with the manufacturer's instructions. The Aβ concentrations were determined by comparison with the standard curve.

### ELISA quantification of estradiol

Three right hippocampus per group for the assay. To measure the levels of estradiol in hippocampi lysates and serum, the rat hippocampi were homogenized in 1× PBS and blood is obtained from the orbital vessels, then centrifuged at 1,500 r for 20 min. Estradiol was quantified using the rat estradiol ELISA Kit (CZVV, Nanjing, China). The results were expressed in ng/L.

### Statistical analysis

Data are descriptively presented as means ± *SD* and analyzed by SPSS 17.0. Statistical analysis was performed using either Student's *t*-test (two-group comparison) for behavior test, dendritic plasticity, Western blot, enzymatic activity. For the levels of estradiol in serum and hippocampus, we firstly performed a descriptive analysis in Supplementary Tables 2, 3, and then a Shapiro–wilk test for a normal distribution of the samples from four group, finally a general linear model to be used for two-way ANOVA followed by *post-hoc* comparison, and differences with *P* < 0.05 were considered significant.

## Results

### Sex influences spatial learning and memory deficits in sporadic AD animal model induced by ICV-STZ

**Figure 1:**
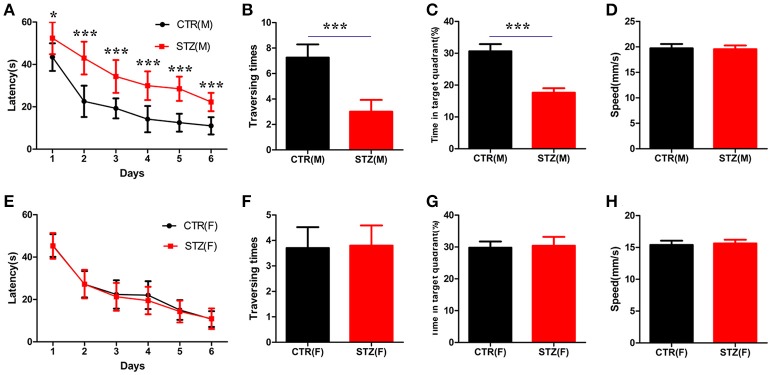
Sex influences spatial learning and memory deficits in sporadic AD animal model induced by ICV-STZ. **(A,E)** Escape latencies to find the hidden platform for male or female rats. **(B,F)** The time spent in the target quadrant and **(C,G)** the crossed times after removing the platform. **(D,H)** The speed of male or female rats. Males are top figures **(A–D)**, females bottom figures **(E–H)**. The data were expressed as mean ± *SD* (*n* = 12). ^***^P < 0.001 vs. the vehicle control. Data were analyzed using *t*-test. ^*^P < 0.05 vs. the vehicle control.

A study showed that a significant cognitive impairment was evoked at the 2nd week onwards, which persisted up to the 14th week with ICV-STZ (3 mg/kg) in rats (Mehla et al., 2013). To investigate whether sex differences exist in cognitive deficits induced by STZ, in the present study, we performed morris water maze to evaluate the memory and learning abilities of rats 30 days after ICV-STZ treatment. For male rats, we found that the escape latency to find a hidden platform dramatically increased while the traversing times and the time in the target quadrant were significantly decreased at the 7th day in ICV-STZ rats when compared to vehicle control (Figures 1A–C). This confirmed that ICV-STZ induced learning and memory deficits in male rats. For female rats, to our surprise, we failed to observe any learning and/or memory deficits. The latency to find the hidden platform, the crossing numbers and time spent in the target quadrant also did not change in female rats (Figures 1E–G). Both groups in male or female rats exhibited comparable swimming speed (Figures 1D,H), indicating that motor function was not affected. Altogether, the findings suggest that ICV-STZ injection induces cognitive impairments in male but not female rats.

### Sex influences loss of dendritic and synaptic plasticity in the sporadic AD animal model induced by ICV-STZ

**Figure 2:**
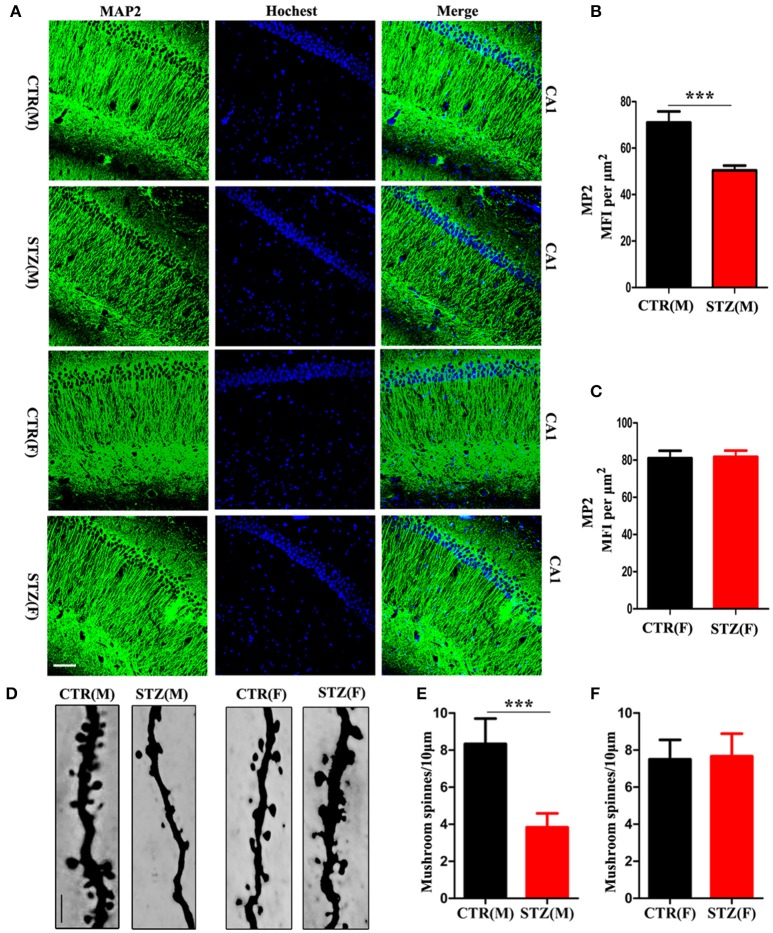
Sex influences loss of dendritic plasticity in sporadic AD animal model. **(A)** MAP2 and DAPI co-staining in the hippocampal CA1 region for male or female rats. Scale bar = 100 μm. **(B,C)** Quantification of MAP2 immunofluorescence. The data were expressed as mean ± *SD* (*n* = 3). **(D)** Representative photomicrographs of dendritic spines in the hippocampal CA1 region. Scale bar = 5 μm. **(E,F)** Quantification of mushroom-type dendritic spines. The data were expressed as mean ± *SD* (*n* = 3). ^***^P < 0.001 vs. the vehicle control. Data were analyzed using *t*-test.

Dendrite complexity (Li et al., 2008) and synaptic plasticity (Kasai et al., 2010) are neurobiological basis for learning and memory. We determined the effect of ICV-STZ on neuronal integrity, by examining levels of the dendritic marker MAP2. For male rats, the semi quantitative results showed a strongly reduced mean fluorescence intensities (MFIs) of MAP2 immunoreactivity in the pyramidal neurons of CA1 region of the hippocampus in ICV-STZ rats compared to vehicle control (Figures 2A,B). However, in female rats, MAP2 immunoreactivity showed that ICV-STZ had no effect on dendritic number compared to control (Figures 2A,C). We also examined alterations in dendritic spines using Golgi staining. Mushroom-type spines in the CA1 of ICV-STZ treated male rats decreased remarkably compared to control (Figures 2D,E), but the number of mushroom-type dendritic spines were not altered in the ICV-STZ treated female rats compared to control (Figures 2D,F).

**Figure 3:**
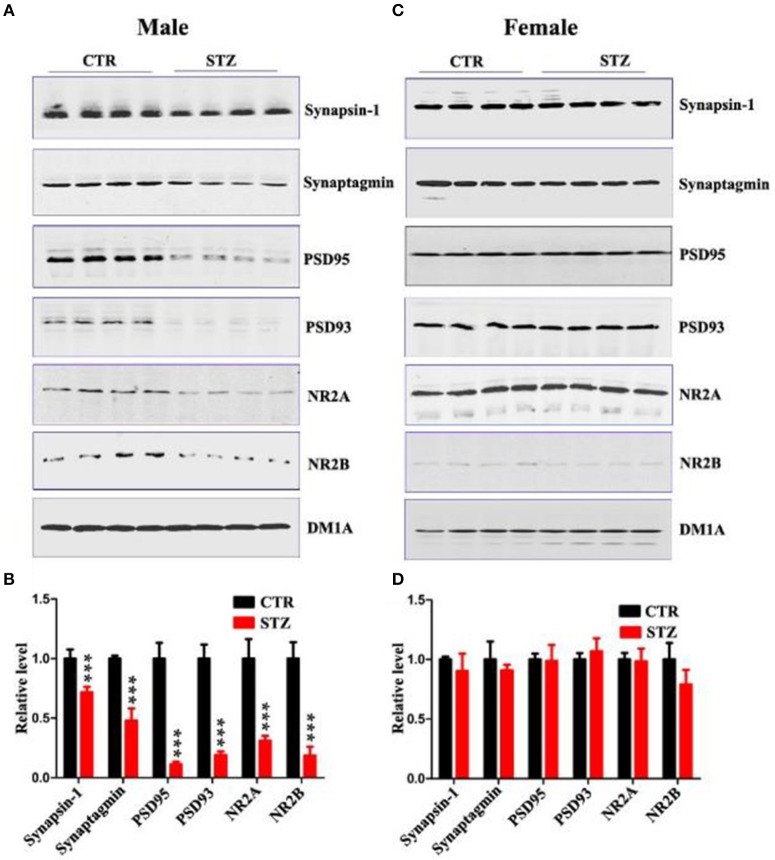
Sex influences synaptic plasticity in the sporadic AD animal model. **(A,C)** Western blot analysis of the protein levels of synapsin-1, synaptagmin, psd95, psd93, NR2A, and NR2B and **(B,D)** their quantitative analysis for male or female rats. DM1A was used as a loading control. The data were expressed as mean ± *SD* (*n* = 4). ^***^P < 0.001 vs. the vehicle control. Data were analyzed using *t*-test.

Normal synaptic function is contingent upon the stable expression of synaptic proteins. Therefore, we evaluated several key synapse-associated proteins using Western blotting. ICV-STZ treatment remarkably suppressed the expressions of presynaptic synapsin I, synaptagmin and postsynaptic PSD95, PSD93, NR2A, and NR2B in male rats (Figures 3A,B). Nonetheless, there is no any significant difference between vehicle and ICV-STZ treated female rats (Figures 3C,D). These data demonstrate that ICV-STZ induces loss of dendritic and synaptic plasticity in male, but not in female rats.

### Sex influences Tau hyperphosphorylation and GSK-3β activity in the sporadic AD animal model induced by ICV-STZ

**Figure 4:**
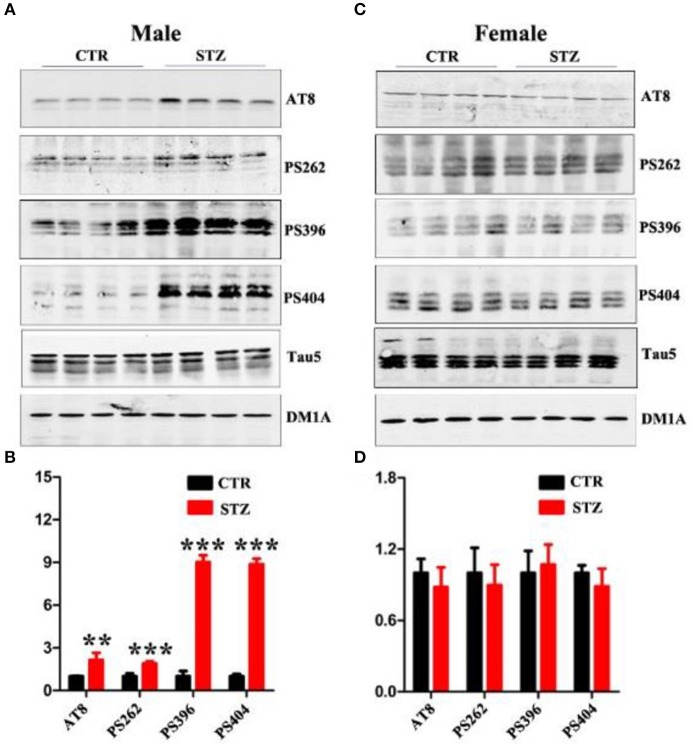
Sex influences tau hyperphosphorylation in the sporadic AD animal model. **(A,C)** Western blot analysis of the protein levels of AT8, PS262, PS396, PS404, and Tau5 and **(B,D)** their quantitative analysis for male or female rats. The data were expressed as mean ± *SD* (*n* = 4). The phosphorylation level of tau was normalized to total tau level probed by tau5. The total level of tau was normalized DM1A. ^***^P < 0.001 vs. the vehicle control. Data were analyzed using *t*-test. ^**^P < 0.01 vs. the vehicle control.

Abnormal hyperphosphorylation and accumulation of Tau play a key role in AD pathology (Wang and Liu, 2008), and hyperphosphorylated tau causes dendritic loss and neurodegeneration (Wang et al., 2010). In addition, ICV-STZ treatment induces tau hyperphosphorylation in rats (Zhou et al., 2013). In this study, we also explored whether ICV-STZ induces tau hyperphosphorylation in male or female rats, respectively. We detected a significantly increasing tau phosphorylation at the Ser199/202(AT8), Ser262, Ser396, and Ser404 sites in ICV-STZ treated male rats (Figures 4A,B). Conversely, in the female rats, ICV-STZ did not induce tau hyperphosphorylation (Figures 4C,D).

**Figure 5:**
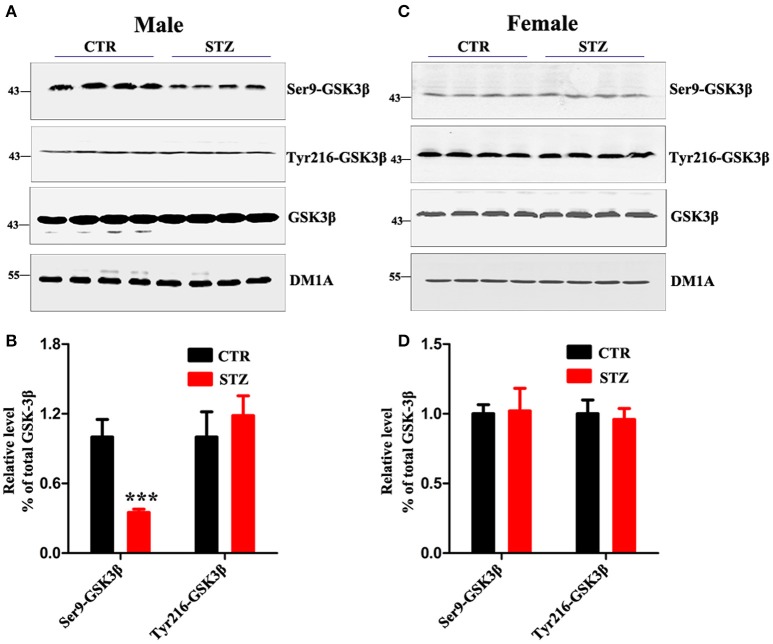
Sex influences activity of GSK-3β in the sporadic AD animal model. **(A,C)** The total GSK-3β, GSK-3β (Ser9), GSK-3β (Tyr216) levels in whole hippocampus extracts were measured using Western blotting and **(B,D)** quantitative analysis. The phosphorylation level of GSK-3β was normalized to total GSK-3β level. The total level of GSK-3β was normalized DM1A. The data were expressed as mean ± *SD* (*n* = 4). ^***^P < 0.001 vs. the vehicle control. Data were analyzed using *t*-test.

GSK-3β is the first identified and critical tau kinase (Singh et al., 1995), therefore we evaluated the total level and the activity-dependent phosphorylation of GSK-3β. In male rats, we found that the p-GSK-3β (Ser9) (the inactive form) level was remarkably decreased, while the level of total GSK-3β and p-GSK-3β (Tyr216) (the active form) didn't change (Figures 5A,B). In female rats, no significant difference was observed between ICV-STZ treated and vehicle control (Figures 5C,D). Taken together, these findings suggest that ICV-STZ activates GSK-3β and consequently leads to hyperphosphorylation of tau protein in male rats, but does not elicit these demonstrable pathological alterations in female rats.

### Sex influences the activity of BACE1 and Aβ production in the sporadic AD animal model induced by ICV-STZ

**Figure 6:**
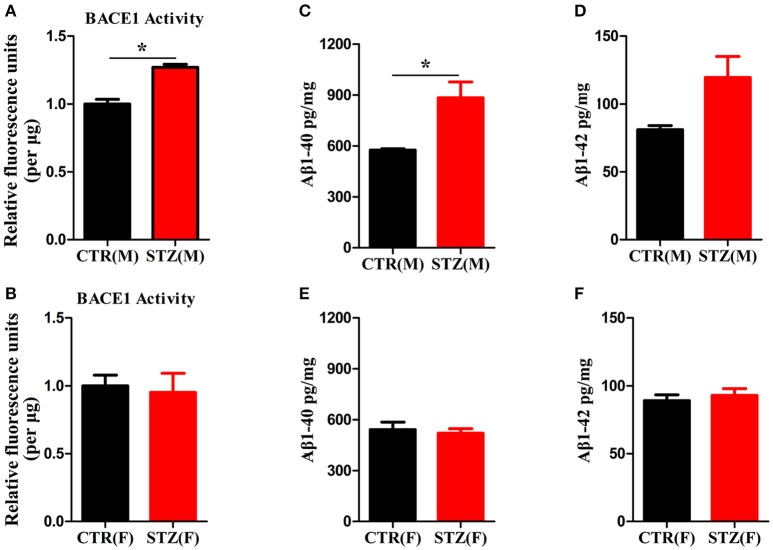
Sex influences activity of BACE1 and Aβ production in the sporadic AD animal model. **(A,B)** BACE1 activity was determined using β-Secretase Activity Assay Kit. **(C–F)** Aβ40/42 levels were quantified through ELISA. The data were expressed as mean ± *SD* (*n* = 5). ^*^P < 0.05 vs. the vehicle group. Data were analyzed using *t*-test.

Another characterized histology of AD is extracellular senile plaques, which are composed of aggregated protein Aβ initiated by β-secretase (BACE1) (Alafuzoff et al., 1987; Vassar et al., 2009). We employed β-secretase Activity Assay Kit and Aβ40/42 Elisa Kit to detect BACE1 activity and Aβ levels of hippocampus. In ICV-STZ treated male rats, both BACE1 activity and Aβ40 level were significantly increased compared to control rats, while Aβ42 showed ascendant trend without significant difference (Figures 6A,C,D). However, in female rats, BACE1 activity and Aβ40/42 levels were not altered in both ICV-STZ and vehicle treated rats (Figures 6B,E,F). Thus, these data strongly support that ICV-STZ increases BACE1 activity and augments Aβ production in male rats, while did not exhibit these toxic effects in female rats.

### Estradiol levels in serum and hippocampus of ICV-STZ treated male and female rats

**Figure 7:**
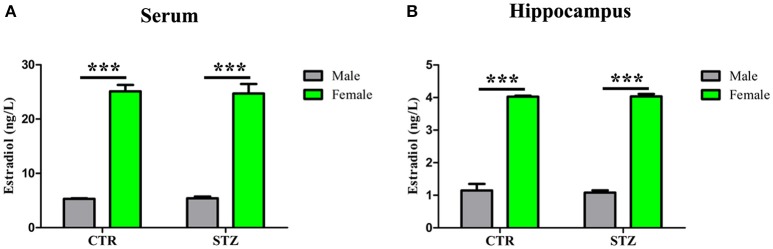
Estradiol levels in serum and hippocampus. The Estradiol levels in the serum **(A)** and hippocampus **(B)** were measured. Estradiol levels of female rats were much higher than that of male rats, while no difference was observed between the groups with same sex. Data were presented as means ± *SD* (*n* = 3). ^***^P < 0.001 vs. male rats. Data were analyzed using a general linear model was used for two-way ANOVA followed by *post-hoc* comparison.

Previous studies have shown that estradiol reduces Aβ production via reducing total BACE1 activity, and decreases tau hyperphosphorylation by mediated GSK-3β activity (Singh et al., 1999; Zhang et al., 2008). To investigate whether estradiol influences the generation of the sporadic AD animal model induced by ICV-STZ, we measured estradiol levels in serum and hippocampus of ICV-STZ treated male and female rats. Shapiro–wilk test showed that all of the *p*-values were > 0.05, indicating estradiol levels in serum (Supplementary Table 4) and hippocampus (Supplementary Table 5) according with normal distribution from each group. And then, a general linear model was used for two-way ANOVA (Supplementary Tables 6, 7) followed by *post-hoc* comparison. We found that estradiol levels of serum (Figure 7A) and hippocampus (Figure 7B) of female rats were much higher than that of male rats, while no difference was observed between the groups with same sex. The data suggest that high estradiol might protect from STZ induced neurotoxic effects in female rats.

## Discussion

Nowadays, AD is a major public health problem, which has been considered as a multifactorial disease associated with several etiopathogenic mechanisms (Iqbal and Grundke-Iqbal, 2010). The first step for a rational drug design is to study etiopathogenic mechanisms and to develop animal models based on these mechanisms. The late-onset sporadic form of AD, which mechanisms still remain unclear due to its multi-etiopathological factors, accounts for over 95% of all cases. However, few experimental animal model of sporadic AD badly limit the studies on its pathogenesis and drug development (Agrawal et al., 2011; Iqbal et al., 2013).

The majority of current animal models of AD are generated as familial one, which express human genes mutations, such as Aβ and tau related gene manipulation. However, animal model of familial AD cannot sufficiently exhibit all pathological alterations and processes (Chen et al., 2013). Therefore, experimental models that faithfully mimic the pathology of sAD are essential to study its mechanism and assess the effectiveness of the therapeutic strategies. Previous research has showed that sAD is being recognized as an insulin resistant brains state (Valente et al., 2010; Bitel et al., 2012; Kamat et al., 2016). Therefore, a non-transgenic animal model generated by ICV-STZ has been proposed as a representative model of sAD. The ICV-STZ rats develop insulin resistant brains state associated with sAD like neuropathological changes and memory impairment (Carro and Torres-Aleman, 2004; Valente et al., 2010; Agrawal et al., 2011; Bitel et al., 2012; Chen et al., 2013; Iqbal et al., 2013; Kamat et al., 2016). Although, the mechanisms underlying ICV-STZ evoked AD pathology remain unknown, ICV-STZ rats have been used in many labs as an experimental model of sAD. For more than 20 years, although some of therapeutic strategies displayed very good effectiveness for AD in ICV-STZ animal models, the same therapies were hard to be reproduced on memory deficits in clinical trials with sAD patients (Salkovic-Petrisic et al., 2013). Thus, it is necessary to re-evaluate the ICV-STZ animal model once again.

Sex has a regulatory effect on brain functions (Brinton, 2009; Cui et al., 2013). We here investigated sex differences on cognitive deficits in the sporadic AD animal model induced by ICV-STZ. Similar to previous studies, the ICV administration of STZ induced cognitive deficits and loss of synaptic plasticity in male rats, but these neurotoxic effects were not observed in female rats. Thus, the ICV-STZ is only for generating animal model of sAD in male, but not in female rats. Consequently sex differences should be considered in AD researches in the future.

Estrogen reduces Aβ level by down-regulating total β secretase activity through MARK/ERK pathway, and modulates Aβ degradation (Pike, 1999; Singh et al., 1999; Vassar et al., 2009). In the present study, we found that ICV-STZ increased BACE1 activities and Aβ40/42 production in male rats, but these alterations were not observed in female rats. The studies have demonstrated that the number of NFTs consisting of hyperphosphorylated tau is positively correlated with the degree of clinical dementia (Iqbal and Grundke-Iqbal, 1991; Iqbal et al., 2008; Luo et al., 2014). Estrogens attenuate tau hperphosphorylation through kinases and phosphatases, such as the GSK-3β, Wnt, and PKA pathways (Zhang et al., 2008). The ICV-STZ model in male rats shows hyperphosphorylation of tau and an increase of GSK-3β activity, but these tau pathologies are not observed in female rats. Together, sex hormones might account for functional discrepancy of ICV-STZ in the two sexes.

A large body of evidence shows that women have a higher incidence of AD than men happening after menopause, which suggest that estrogen might protect against AD pathology. Hormones have long been known to play key roles in regulating learning and memory and ample evidence has demonstrated that estradiol affects hippocampal morphology, plasticity, and memory (Packard, 1998; Brinton, 2009; Foster, 2012; Cui et al., 2013). Studies in the aromatase knock-out mouse suggest that estradiol induced spine and spine synapse formation in hippocampus, not in the cortex or the cerebellum (Zhou et al., 2014). Previous studies point to a role of hippocampus-derived estradiol in synaptic plasticity in cultured slices and *in vivo*, not just the role of gonads-derived estradiol (Zhou et al., 2010; Vierk et al., 2012). In addition, dendritic spines of CA1 pyramidal neurons vary during estrus cyclicity, which likely results from cycle of estradiol synthesis in the hippocampus, since gonadotropin releasing hormone regulates estradiol synthesis in the hippocampus in a dose-dependent manner (Woolley et al., 1990; Prange-Kiel et al., 2008, 2013). In the present study, 10 rats employed in each group is a small sample size. Therefore, we performed Shapiro–wilk test which showed that samples from each group was in accord with normal distribution. Relatively we found that hippocampal estradiol level of female rats is almost four times higher than that of male rats in both vehicle and ICV-STZ treated groups, which is in accordance with previous studies by using mass spectrometry (Fester et al., 2012). This implies that high estradiol levels in female rats might protect them from the ICV-STZ induced cognitive deficits and neurodegenerative pathologies, including synaptic damage, Aβ deposition, and tau hyperphosphorylation in hippocampus.

In reported literature, optimal female performance occurred during the phase of estrus on the spatial learning and memory, and the least efficient performance occurred during proestrus (Vina and Lloret, 2010). Since we did not determine the estrus stage of the control animals and cognitive ability and synaptic density are optimal during proestrus, the estradiol protective effects on hippocampal plasticity and memory would very likely have been greater if we had had exclusively taken proestrus female rats.

Although, the mouse- and monkey ICV-STZ models have also been developed, ICV-STZ rats are still widely used and employed to evaluate the therapeutic potential of drugs and non-drug therapies in numerous laboratories. Cognitive deficits and AD-like pathology, such as neuroinflammation, brain insulin resistance, tau hyperphosphorylation, Aβ overproduction, have been found both in female mice and monkeys (Chen et al., 2014; Park et al., 2015). Liu et al. have reported that STZ inhibits the Ras/ERK signaling cascade and decreased the phosphorylation of CREB, and induces cognitive impairment in rats (Liu et al., 2013). However, the study from Diao et al. shows the gender- and EC-dependent levels of proteins from the protein synthetic, chaperoning, and degradation machinery (Diao et al., 2007). Accordingly, it is necessary to re-evaluate the STZ-induced cognitive alterations between male and female rats. In the present study, we found that ICV-STZ remarkably results in cognitive impairments and AD like pathological alterations in the Sprague-Dawley male rats, but not in the female rats. It may conceivably be related with the gender- and EC-dependent levels of proteins from the protein synthetic, chaperoning, and degradation machinery, and consequently regulates tau related kinases and APP cleavage. Its molecular mechanism is worth further discussing. Our findings provide novel insights suggesting that sex differences exist in ICV-STZ rats which have been used as sporadic AD animal model for about 20 years. Therefore, our study encourages investigators to comply with National Institutes of Health policies to include females in biomedical research and to be aware that adding females to a study is not as simple as adding just another group.

## Author contributions

XW and JW designed the experiments. JB performed the experiments and analyzed data. JB, YM, BZ, RL, ZZ, JW, and XW discussed and interpreted the results. JB, YM, and XW wrote the paper. All authors have approved the final version of the manuscript.

### Conflict of interest statement

The authors declare that the research was conducted in the absence of any commercial or financial relationships that could be construed as a potential conflict of interest.

## Supplementary material

Supplementary Figure1 The experiments were designed as shown above.
