# Corrigendum: Sex Differences in the Cognitive and Hippocampal Effects of Streptozotocin in an Animal Model of Sporadic AD

**DOI:** 10.3389/fnagi.2019.00275

**Published:** 2019-10-15

**Authors:** Jian Bao, Yacoubou A. R. Mahaman, Rong Liu, Jian-Zhi Wang, Zhiguo Zhang, Bin Zhang, Xiaochuan Wang

**Affiliations:** ^1^Key Laboratory of Ministry of Education of China for Neurological Disorders, Department of Pathophysiology, School of Basic Medicine and the Collaborative Innovation Center for Brain Science, Tongji Medical College, Huazhong University of Science and Technology, Wuhan, China; ^2^Co-innovation Center of Neuroregeneration, Nantong University, Nantong, China; ^3^School of Medicine and Health Management, Tongji Medical College, Huazhong University of Science and Technology, Wuhan, China; ^4^Department of Genetics and Genomic Sciences, Icahn School of Medicine at Mount Sinai, New York, NY, United States

**Keywords:** Alzheimer's disease (AD), animal model, Streptozotocin (STZ), sex differences, learning and memory

In the original article, due to the authors' oversight, there were several mistakes in Figure 3C and Figure 4C as published. The image “PSD95 of Female” in Figure 3C was inadvertently replaced with the image “GSK3β” in Figure 5C. The images “AT8 of Female” and “PS262 of Female” in Figure 4C were inadvertently replaced with image “PS404 of Female” from Figure 4C. The image “Tau5 of Female” in Figure 4C was inadvertently replaced with image “Tau5 of Male” in Figure 4A. The corrected Figures 3C and 4C appear below. The quantification for the above-mentioned blots have been done in the corrected Figures 3D and 4D, which all have no significant difference between control and STZ groups as in the published original version of the article.

**Figure 3:**
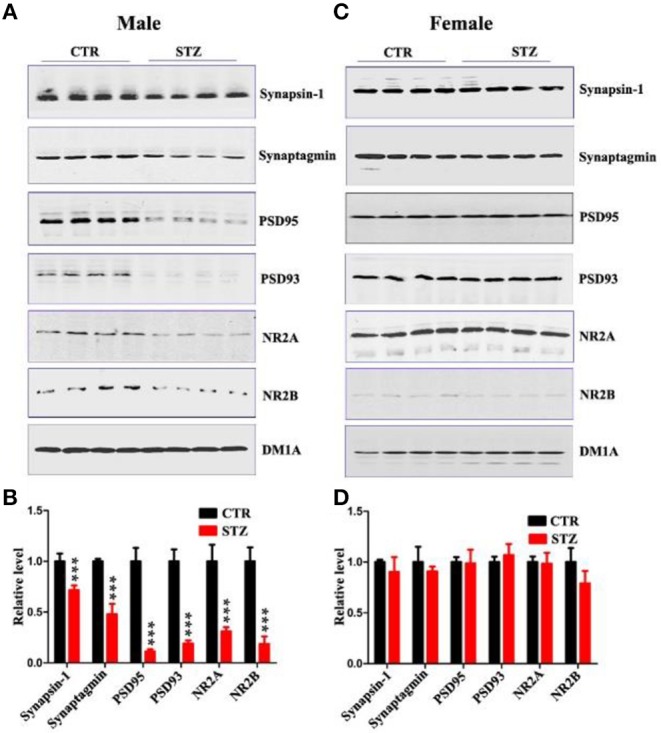
Sex influences synaptic plasticity in the sporadic AD animal model. **(A,C)** Western blot analysis of the protein levels of synapsin-1, synaptagmin, psd95, psd93, NR2A, and NR2B and **(B,D)** their quantitative analysis for male or female rats. DM1A was used as a loading control. The data were expressed as mean ± *SD* (*n* = 4). ***P < 0.001 vs. the vehicle control. Data were analyzed using *t*-test.

**Figure 4:**
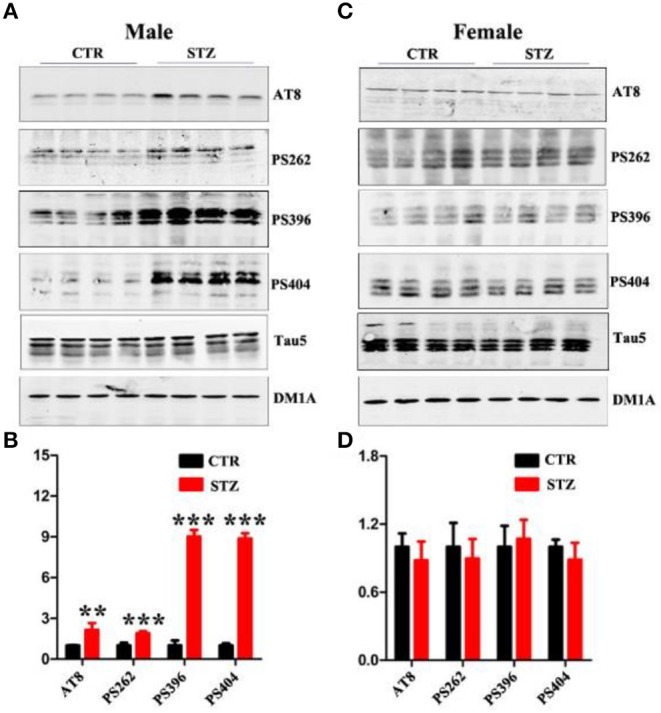
Sex influences tau hyperphosphorylation in the sporadic AD animal model. **(A,C)** Western blot analysis of the protein levels of AT8, PS262, PS396, PS404, and Tau5 and **(B,D)** their quantitative analysis for male or female rats. The data were expressed as mean ± *SD* (*n* = 4). The phosphorylation level of tau was normalized to total tau level probed by tau5. The total level of tau was normalized DM1A. ***P < 0.001 vs. the vehicle control. Data were analyzed using *t*-test. **P < 0.01 vs. the vehicle control.

The authors apologize for this error and state that this does not change the scientific conclusions of the article in any way. The original article has been updated.

