# Supplementary figures and images for: Sex Differences in the Cognitive and Hippocampal Effects of Streptozotocin in an Animal Model of Sporadic AD
Source: Front Aging Neurosci. 2017 Oct 31;9:347. doi: 10.3389/fnagi.2017.00347 (PMC5671606; doi:10.3389/fnagi.2017.00347)

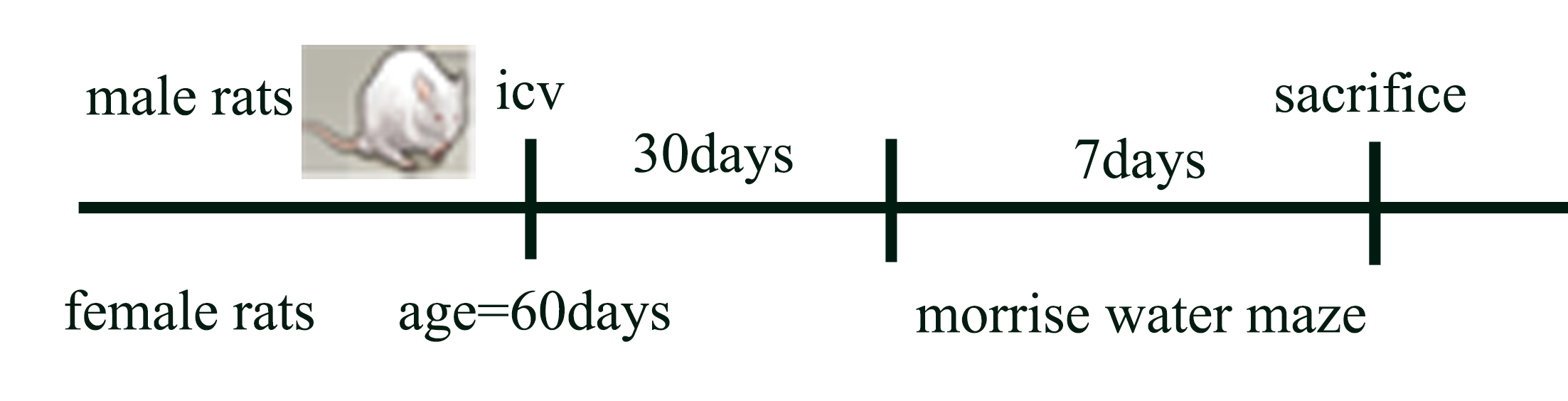

Supplement: Supplementary Figure1 — The experiments were designed as shown above. [file Image1.TIF]
