# Supplementary material for: Sex Differences in the Cognitive and Hippocampal Effects of Streptozotocin in an Animal Model of Sporadic AD
Source: Front Aging Neurosci. 2017 Oct 31;9:347. doi: 10.3389/fnagi.2017.00347 (PMC5671606; doi:10.3389/fnagi.2017.00347)
Supplement: Supplementary file 2 [file Table1.DOCX]

**Supplementary Table 1. Antibodies employed in this study**

| **Antibody** | **Specific** | **Type/host** | **Dilution** | **Source** |
| --- | --- | --- | --- | --- |
| MAP2 | MAP2 | Poly-/Rabbit | 1:200 for IF | Abcam, Cambridge, MA, USA |
| p-GSK-3β(S216) | Phosphorylated GSK-3βat Ser216 | Poly-/ Rabbit | 1:1000 for WB | Cell Signaling Danvers, MA, USA |
| p-GSK-3β(S9) | Phosphorylated GSK-3βat Ser9 | Poly-/ Rabbit | 1:1000 for WB | Cell Signaling Danvers, MA, USA |
| GSK-3β | Total GSK-3β | Mono-/Mouse | 1:1000 for WB | Cell Signaling Danvers, MA, USA |
| Synaptotagmin | Synaptotagmin | Mono-/Mouse | 1:1000 for WB | Millipore, Temecula, CA, USA |
| Synapsin-1 | Synapsin-1 | Mono-/Mouse | 1:1000 for WB | Millipore, Temecula, CA, USA |
| NR2A | NR2A | Mono-/Mouse | 1:1000 for WB | Millipore, Temecula, CA, USA |
| NR2B | NR2B | Mono-/Mouse | 1:1000 for WB | Millipore, Temecula, CA, USA |
| PSD95 | PSD95 | Poly-/ Rabbit | 1:1000 for WB | Abcam, Cambridge, MA, USA |
| PSD93 | PSD93 | Poly-/ Rabbit | 1:1000 for WB | Abcam, Cambridge, MA, USA |
| AT8 | Phosphorylated tau at Ser202/Thr205 | Mono-/Mouse | 1:1000 for WB | Thermo Fisher Scientific, Kalamazoo, MI, USA |
| PS262 | Phosphorylated tau at Ser262 | Poly-/ Rabbit | 1:1000 for WB | Signalway Antibody College Park, MD, USA |
| PS396 | Phosphorylated tau at Ser396 | Poly-/ Rabbit | 1:1000 for WB | Abcam, Cambridge, MA, USA |
| PS404 | Phosphorylated tau at Ser404 | Poly-/ Rabbit | 1:1000 for WB | Signalway Antibody College Park, MD, USA |
| Tau-5 | Total tau | Mono-/Mouse | 1:1000 for WB | Millipore, Temecula, CA, USA |
| DM1A | alpha-tublin | Mono-/Mouse | 1:1000 for WB | Signalway Antibody College Park, MD, USA |

Mono-, monoclonal; poly-, polyclonal; WB, Westernblotting; IF, Immunofluorescence; P, phosphorylated
