# Supplementary material for: Sex Differences in the Cognitive and Hippocampal Effects of Streptozotocin in an Animal Model of Sporadic AD
Source: Front Aging Neurosci. 2017 Oct 31;9:347. doi: 10.3389/fnagi.2017.00347 (PMC5671606; doi:10.3389/fnagi.2017.00347)
Supplement: Supplementary file 3 [file Table2.DOCX]

**Supplementary Table 2. Descriptives for estradiol levels in serum**

| Group | | | Statistic | Std. Error |
| --- | --- | --- | --- | --- |
| Male, STZ | Mean | | 5.4362913 | .13292027 |
|  | 95% Confidence Interval for Mean | Lower Bound | 4.8643816 |  |
|  |  | Upper Bound | 6.0082011 |  |
|  | 5% Trimmed Mean | | . |  |
|  | Median | | 5.4736500 |  |
|  | Variance | | .053 |  |
|  | Std. Deviation | | .23022466 |  |
|  | Minimum | | 5.18967 |  |
|  | Maximum | | 5.64555 |  |
|  | Range | | .45588 |  |
|  | Interquartile Range | | . |  |
|  | Skewness | | -.711 | 1.225 |
|  | Kurtosis | | . | . |
| Male,  CTR | Mean | | 5.3106507 | .04100657 |
|  | 95% Confidence Interval for Mean | Lower Bound | 5.1342136 |  |
|  |  | Upper Bound | 5.4870877 |  |
|  | 5% Trimmed Mean | | . |  |
|  | Median | | 5.3000000 |  |
|  | Variance | | .005 |  |
|  | Std. Deviation | | .07102547 |  |
|  | Minimum | | 5.24555 |  |
|  | Maximum | | 5.38640 |  |
|  | Range | | .14085 |  |
|  | Interquartile Range | | . |  |
|  | Skewness | | .660 | 1.225 |
|  | Kurtosis | | . | . |
| Female,  STZ | Mean | | 24.7208367 | .71798096 |
|  | 95% Confidence Interval for Mean | Lower Bound | 21.6316139 |  |
|  |  | Upper Bound | 27.8100594 |  |
|  | 5% Trimmed Mean | | . |  |
|  | Median | | 24.7523500 |  |
|  | Variance | | 1.546 |  |
|  | Std. Deviation | | 1.24357950 |  |
|  | Minimum | | 23.46180 |  |
|  | Maximum | | 25.94836 |  |
|  | Range | | 2.48656 |  |
|  | Interquartile Range | | . |  |
|  | Skewness | | -.114 | 1.225 |
|  | Kurtosis | | . | . |
| Female,  CTR | Mean | | 24.9779900 | .50466680 |
|  | 95% Confidence Interval for Mean | Lower Bound | 22.8065840 |  |
|  |  | Upper Bound | 27.1493960 |  |
|  | 5% Trimmed Mean | | . |  |
|  | Median | | 24.7333300 |  |
|  | Variance | | .764 |  |
|  | Std. Deviation | | .87410853 |  |
|  | Minimum | | 24.25228 |  |
|  | Maximum | | 25.94836 |  |
|  | Range | | 1.69608 |  |
|  | Interquartile Range | | . |  |
|  | Skewness | | 1.161 | 1.225 |
|  | Kurtosis | | . | . |
