# Supplementary material for: Sex Differences in the Cognitive and Hippocampal Effects of Streptozotocin in an Animal Model of Sporadic AD
Source: Front Aging Neurosci. 2017 Oct 31;9:347. doi: 10.3389/fnagi.2017.00347 (PMC5671606; doi:10.3389/fnagi.2017.00347)
Supplement: Supplementary file 4 [file Table3.DOCX]

**Supplementary Table 3. Descriptives for estradiol levels in hippocampus**

| Group | | | Statistic | Std. Error |
| --- | --- | --- | --- | --- |
| Male, STZ | Mean | | 1.07092333 | .031490175 |
|  | 95% Confidence Interval for Mean | Lower Bound | .93543204 |  |
|  |  | Upper Bound | 1.20641462 |  |
|  | 5% Trimmed Mean | | . |  |
|  | Median | | 1.05255000 |  |
|  | Variance | | .003 |  |
|  | Std. Deviation | | .054542584 |  |
|  | Minimum | | 1.027940 |  |
|  | Maximum | | 1.132280 |  |
|  | Range | | .104340 |  |
|  | Interquartile Range | | . |  |
|  | Skewness | | 1.344 | 1.225 |
|  | Kurtosis | | . | . |
| Male,  CTR | Mean | | 1.11097333 | .088886790 |
|  | 95% Confidence Interval for Mean | Lower Bound | .72852435 |  |
|  |  | Upper Bound | 1.49342232 |  |
|  | 5% Trimmed Mean | | . |  |
|  | Median | | 1.03752000 |  |
|  | Variance | | .024 |  |
|  | Std. Deviation | | .153956436 |  |
|  | Minimum | | 1.007500 |  |
|  | Maximum | | 1.287900 |  |
|  | Range | | .280400 |  |
|  | Interquartile Range | | . |  |
|  | Skewness | | 1.658 | 1.225 |
|  | Kurtosis | | . | . |
| Female,  STZ | Mean | | 4.02751700 | .031481884 |
|  | 95% Confidence Interval for Mean | Lower Bound | 3.89206139 |  |
|  |  | Upper Bound | 4.16297261 |  |
|  | 5% Trimmed Mean | | . |  |
|  | Median | | 4.01000000 |  |
|  | Variance | | .003 |  |
|  | Std. Deviation | | .054528222 |  |
|  | Minimum | | 3.983900 |  |
|  | Maximum | | 4.088651 |  |
|  | Range | | .104751 |  |
|  | Interquartile Range | | . |  |
|  | Skewness | | 1.296 | 1.225 |
|  | Kurtosis | | . | . |
| Female,  CTR | Mean | | 4.02117700 | .013318690 |
|  | 95% Confidence Interval for Mean | Lower Bound | 3.96387130 |  |
|  |  | Upper Bound | 4.07848270 |  |
|  | 5% Trimmed Mean | | . |  |
|  | Median | | 4.01005100 |  |
|  | Variance | | .001 |  |
|  | Std. Deviation | | .023068648 |  |
|  | Minimum | | 4.005780 |  |
|  | Maximum | | 4.047700 |  |
|  | Range | | .041920 |  |
|  | Interquartile Range | | . |  |
|  | Skewness | | 1.665 | 1.225 |
|  | Kurtosis | | . | . |
