# Supplementary material for: Sex Differences in the Cognitive and Hippocampal Effects of Streptozotocin in an Animal Model of Sporadic AD
Source: Front Aging Neurosci. 2017 Oct 31;9:347. doi: 10.3389/fnagi.2017.00347 (PMC5671606; doi:10.3389/fnagi.2017.00347)
Supplement: Supplementary file 6 [file Table5.DOCX]

**Supplementary Table 5.**

**Tests of Normality for estradiol levels in hippocampus from 4 groups**

| Group | Shapiro-Wilk | | |
| --- | --- | --- | --- |
|  | Statistic | df | Sig. |
| Male, STZ | 0.915 | 3 | 0.435 |
| Male, CTR | 0.829 | 3 | 0.186 |
| Female, STZ | 0.923 | 3 | 0.462 |
| Female, CTR | 0.826 | 3 | 0.177 |
