# Supplementary material for: Sex Differences in the Cognitive and Hippocampal Effects of Streptozotocin in an Animal Model of Sporadic AD
Source: Front Aging Neurosci. 2017 Oct 31;9:347. doi: 10.3389/fnagi.2017.00347 (PMC5671606; doi:10.3389/fnagi.2017.00347)
Supplement: Supplementary file 7 [file Table6.DOCX]

**Supplementary Table 6.**

**Two-way ANOVA for estradiol levels in serum**

| Tests of Between-Subjects Effects | | | | | |
| --- | --- | --- | --- | --- | --- |
| Dependent Variable: **estradiol levels** | | | | | |
| Source | Type III Sum of Squares | df | Mean Square | F | Sig. |
| Corrected Model | 1138.060a | 3 | 379.353 | 640.636 | 0.000 |
| Intercept | 2740.268 | 1 | 2740.268 | 4627.652 | 0.000 |
| gender | 1137.937 | 1 | 1137.937 | 1921.701 | 0.000 |
| treat | 0.013 | 1 | 0.013 | 0.022 | 0.886 |
| gender * treat | 0.110 | 1 | 0.110 | 0.186 | 0.678 |
| Error | 4.737 | 8 | 0.592 |  |  |
| Total | 3883.065 | 12 |  |  |  |
| Corrected Total | 1142.797 | 11 |  |  |  |
| a. R Squared =0.996 (Adjusted R Squared =0.994) | | | | | |
