# Supplementary material for: Sex Differences in the Cognitive and Hippocampal Effects of Streptozotocin in an Animal Model of Sporadic AD
Source: Front Aging Neurosci. 2017 Oct 31;9:347. doi: 10.3389/fnagi.2017.00347 (PMC5671606; doi:10.3389/fnagi.2017.00347)
Supplement: Supplementary file 8 [file Table7.DOCX]

**Supplementary Table 7.**

**Two-way ANOVA for estradiol levels in hippocampus**

| Tests of Between-Subjects Effects | | | | | |
| --- | --- | --- | --- | --- | --- |
| Dependent Variable: **estradiol levels** | | | | | |
| Source | Type III Sum of Squares | df | Mean Square | F | Sig. |
| Corrected Model | 25.817a | 3 | 8.606 | 1140.464 | 0.000 |
| Intercept | 78.499 | 1 | 78.499 | 10403.051 | 0.000 |
| gender | 25.814 | 1 | 25.814 | 3421.066 | 0.000 |
| treat | 0.001 | 1 | 0.001 | 0.113 | 0.745 |
| gender * treat | 0.002 | 1 | 0.002 | 0.214 | 0.656 |
| Error | 0.060 | 8 | 0.008 |  |  |
| Total | 104.376 | 12 |  |  |  |
| Corrected Total | 25.877 | 11 |  |  |  |
| a. R Squared =0.998 (Adjusted R Squared =0.997) | | | | | |
